# Kallikrein-related peptidase 5 induces miRNA-mediated anti-oncogenic pathways in breast cancer

**DOI:** 10.18632/oncoscience.91

**Published:** 2014-10-24

**Authors:** Konstantinos G. Sidiropoulos, Nicole M.A. White, Anna Bui, Qiang Ding, Peter Boulos, Georgios Pampalakis, Heba Khella, Joseph N. Samuel, Georgia Sotiropoulou, George M. Yousef

**Affiliations:** ^1^ The Keenan Research Center for Biomedical Sciences at the Li Ka Shing Knowledge Institute and Department of Laboratory Medicine, St. Michael's Hospital, Toronto, Canada; ^2^ Department of Laboratory Medicine and Pathobiology, University of Toronto, Toronto, Canada; ^3^ Department of Pharmacy, School of Health Sciences, University of Patras, Rion-Patras, Greece

**Keywords:** Kallikrein-related peptidase, KLK5, breast cancer, personalized medicine, miRNA, tumour markers

## Abstract

Kallikrein-related peptidase 5 (KLK5) displays aberrant expression in cancer. Recently, we showed KLK5 reconstitution in breast cancer cell lines suppresses malignancy. Present study aims to investigate the functional KLK5 mediated miRNA network on breast cancer progression, molecular subtype and survival.

28 miRNAs were up-regulated and 62 miRNAs were down-regulated upon KLK5 expression. Extracellular matrix (ECM) molecules and cell-adhesion pathways were the most significant KLK5-induced miRNA-mediated regulatory targets. Validation from The Cancer Genome Atlas (TCGA) database indicated KLK5 was specifically down-regulated in luminal B and basal-like breast cancer subtypes. There was a correlation between KLK5, miRNAs and their downstream ECM gene targets. Long-term patient survival correlated with dysregulation of KLK5 and interacting ECM target genes. It suggests biological differences between breast cancer molecular subtypes, patient survival, and their propensity for invasion and metastasis can be explained in part by altered miRNA networks induced by KLK5 dysregulation.

We provide the first evidence that KLK5 can affect miRNA networks, which regulate MMPs and other novel ECM targets and a new compelling hypothesis of interplay between serine proteases and miRNAs. We developed a combined KLK5-(ITGB1+COL12A1) predictive score for recurrence-free survival that could be exploited in clinical applications.

## INTRODUCTION

Breast cancer is the most common cancer and the leading cause of death of cancer for women [1]. In addition to the histological subtypes, it is now clear that distinct “biological” and molecular subtypes associate with vastly different clinical outcomes [2,3]. An understanding of the mechanisms that influence tumor behavior is key to an era of personalized medicine [4].

The human kallikrein-related peptidases (KLK) are expressed in various tissues including breast, ovary, prostate, and kidney [5]. They have been found to be dysregulated in different cancers [6]. KLK5 has been reported to be dysregulated in breast cancer with its over-expression is indicative of good prognosis [7,8,9,10]. We recently showed that KLK5 exerts tumour suppressive effects by inhibiting the mevalonate pathway in breast cancer cells, thus, inhibiting the activation of signaling oncoproteins due to inhibited prenylation [11]. Nonetheless, other molecular mechanisms by which KLK5 influences tumour behaviour are yet to be elucidated.

MicroRNAs (miRNAs) are 19-25-nucleotide, non-coding RNAs, which negatively regulate gene expression post-transcriptionally [12]. miRNAs are dysregulated in many cancers and, depending on their target, can act as oncogenes or tumor suppressors [13]. Recent literature indicate the presence of miRNA networks that can synergistically control cellular pathways [14]. The regulation of KLK by miRNAs was proposed recently [15,16], and demonstrated in kidney, prostate and ovarian cancers [17,18,19]. However, the effect of KLKs on miRNA expression has not been elucidated.

Therefore, we investigated KLK5-miRNA networks of interaction and their potential effects on the pathogenesis of breast cancer. We show for the first time that KLK5 can affect breast cancer pathogenesis through miRNA-mediated pathways. We analyzed the role of miRNA as downstream effectors of KLK5 through identification of a network of miRNAs that are dysregulated in response to altering KLK5 expression. Target prediction, pathway analyses, experimental validation, and clinical data analyses indicate that KLK5 regulates miRNA networks involved in divergent and convergent miRNA pathways that regulate extracellular matrix (ECM) molecules. In addition, we provide evidence that KLK5 can affect the miRNA biogenesis machinery. Importantly, our clinical data validation demonstrates that the miRNA-KLK-ECM interaction matrix not only contributes to the different phenotypic and molecular sub-groups of breast cancer but is also likely to be of benefit for prognostic and therapeutic intervention.

## RESULTS

### KLK5 affects miRNA expression

**Figure 1:**
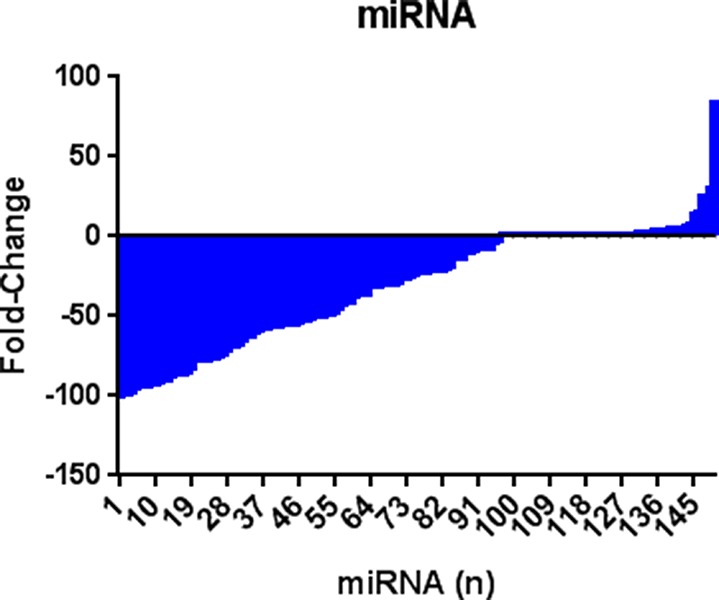
KLK5 overexpression induces miRNA differential expression 28 miRNAs were found to be significantly up-regulated and 62 miRNAs were down-regulated (at least +/− 0.5 fold) in KLK5 stably transfected cells compared to mock transfected cells. Mean of −30.67 fold reduction (SD = 36.13, SEM 2.950). 10% Percentile = −86.72 fold reduction; 90% Percentile= +3.602 fold increase.

The MDA-MB-231 invasive breast cancer cell line was stably transfected with *KLK5* cDNA to express physiological levels of the KLK5 mRNA and protein. Two clones were used for analysis. We compared the expression levels of 754 human miRNAs between the *KLK5-*transfected cells and their parental and vector-transfected (mock) counterparts. We identified 28 miRNAs that were up-regulated and 62 miRNAs that were down-regulated by more than 0.5-fold upon KLK5 expression. These included up-regulated miR-183-5p, miR-206, miR-181-c, and miR-19a and down-regulated miR-935, miR-519a-5p, and miR-23b-5p. The top 10 up- and down-regulated miRNAs are shown in Table 1. Overall, there was a significant trend of miRNA down-regulation with KLK5 expression with a mean of −30.67 fold reduction (SD = 36.13, SEM = 2.950). 10% Percentile = −86.72 fold reduction; 90% Percentile= +3.602 fold increase (Figure 1).

**Table 1:** The top 10 up and down-regulated miRNAs in MDA-MB-231 cells stably transfected with KLK5 compared to controls

Up-regulated miRNA		Down-regulated miRNA	
miRNA	Fold Change	miRNA	Fold Change
hsa-miR-183-5p	83.4	hsa-miR-935	−225.3
hsa-miR-206	30.4	hsa-miR-519a-3p	−102.1
hsa-miR-181c-5p	25.6	hsa-miR-23b-5p	−64.6
hsa-miR-19a-5p	25.0	hsa-miR-888-5p	−48.3
hsa-miR-449b-5p	15.0	hsa-miR-10b-5p	−20.1
hsa-miR-1301	13.5	hsa-miR-520d-3p	−17.3
hsa-let-7a-3p	8.0	hsa-miR-1255b-5p	−15.0
hsa-miR-25-5p	6.3	hsa-miR-545-5p	−13.3
hsa-miR-335-3p	5.2	hsa-miR-650	−12.7
hsa-miR-335-5p	4.7	hsa-miR-342-3p	−10.8

### Target prediction and pathway analyses

The most significant predicted gene targets of the top dysregulated miRNAs are shown in Table 2. We experimentally validated the correlation between the expression of these targets and their targeting miRNAs by comparing gene expression profiles before and after KLK5 transfection using microarray analysis. As shown in Table 2, many of these genes were found to have an inverse correlation in expression changes with their targeting miRNA upon KLK5 transfection. In order to elucidate the involvement of these targets in cancer pathogenesis, we performed pathway and gene enrichment analysis for all targets (Supplementary Table 1 and Supplementary Table 2). KLK5 expression induced down-regulation of a set of miRNAs identified as enriched in the MAPK signaling, apoptosis and p53 pathways. It also caused up-regulation of a set of miRNAs that is associated with GnRH signaling, and ECM-receptor interaction pathways.

**Table 2:** Differential expression of the predicted targets of the top dysregulated miRNAs in MDA-MB-231 cell lines with KLK5 reconstituted expression

Up-regulated miRNA		Gene mRNA Target in Homo sapiens		Down-regulated miRNA		Gene mRNA Target in Homo sapiens	
miRNA	Fold Change*	Symbol	mRNA Dysregulation**	miRNA	Fold Change*	Symbol	mRNA Dysregulation**
miR-183-5p	83.4	CX3CL1	−3.85	miR-935	−225.3	TNRC6B	1.48
		TUB	−2.13			DNMT3B	1.59
		RPS6KA3	−1.56			ARL3	1.65
miR-206	30.41	DNAL1	−2			HMGB1	1.77
		MPZL1	−1.89			MBNL2	1.82
		NMT2	−1.69	miR-519a-3p	−102.1	FMNL3	1.46
		SLC7A6	−1.59			TNRC6B	1.48
		HIGD1A	−1.54			C1orf63	1.53
		HSPD1	−1.47			GLS	1.61
		DLG4	−1.47			ZFHX4	1.64
		G3BP2	−1.47			SLC25A23	1.64
		POLA1	−1.45			ATM	1.75
		PLCG1	−1.45			SORL1	1.83
miR-181c-5p	25.59	CYP26B1	−2.13			LDLR	1.97
		TUB	−2.13			ZHX2	2.31
		DNAL1	−2			FILIP1L	3.69
		FGFR3	−1.89	miR-888-5p	−48.29	KIF1B	1.6
		RASSF2	−.185			DEPDC1B	1.68
		NMT2	−1.69			SORL1	1.83
		ZNF594	−1.67			ZHX2	2.31
		RPS6KA3	−1.56			KCTD12	2.36
		G3BP2	−1.47	miR-10b-5p	−20.12	TNRC6B	1.48
		POLA1	−1.45			RBMS3	1.81
miR-449b-5p	14.99	LMTK3	−3.57			SIX4	1.83
		SYNJ1	−1.75			STARD13	2.01
		NMT2	−1.69			NAV1	2.28
		SLC7A6	−1.59	miR-520d-3p	−17.312	MRPS25	1.44
		PLCG1	−1.45			FMNL3	1.46
		C16orf5	−1.43			TNRC6B	1.48
miR-1301	13.5	NBL1	−1.67			ARHGEF18	1.49
		G3BP2	−1.47			RAB22A	1.59
miR-335-5p	4.742424	NMT2	−1.69			GLS	1.61
						PDCD4	1.64
						ZFHX4	1.64
						FNDC3A	1.68
						SLC35E1	1.82
						MBNL2	1.82
				miR-1255b-5p	−14.962	NAV1	2.28
						FILIP1L	3.69
				miR-650	−12.622	TNRC6B	1.48
						ZFHX4	1.64
						TRAF1	1.69
						SORL1	1.83
						SCD	4.99
				miR-342-3p	−10.714	DLGAP4	1.59
						ZFHX4	1.64
						SORL1	1.83

* Assessed by qRT-PCR screening

** Assessed by Illumina Chip

**Figure 2:**
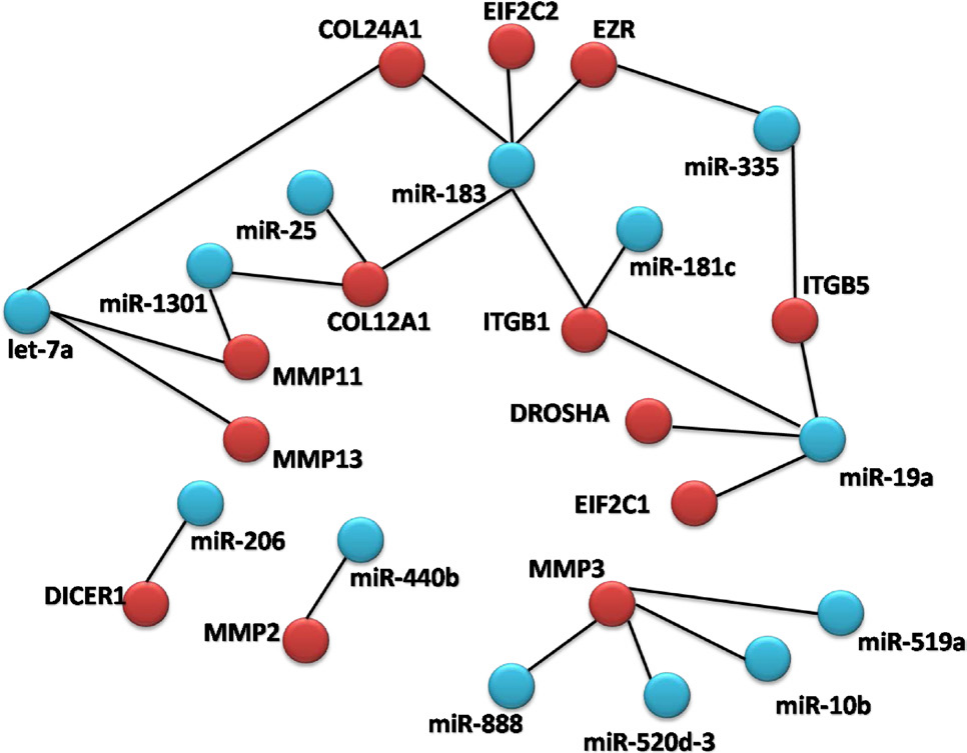
miRNA network diagram Diagram demonstrating miRNA networks with convergent (where multiple miRNAs can target the same gene) and divergent (where the same miRNA can target multiple genes along the same pathway) properties.

**Figure 3:**
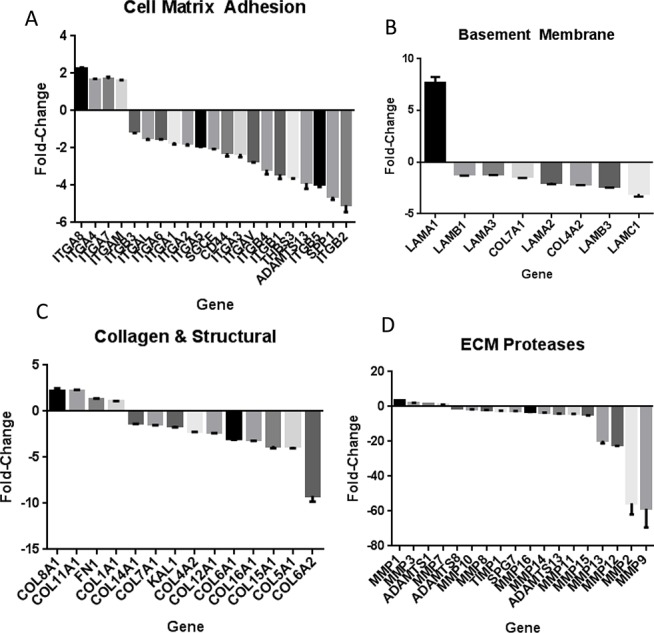
(A)ECM array analysis of cell-matrix adhesion molecules A panel of cell-matrix proteins was analyzed for mRNA expression changes with KLK5 stable transfection compared to mock using an RT Profiler Array. There was a generalized down-regulation of cell-matrix adhesion molecule mRNA expression with KLK5 including the integrin family (p < 0.001).(B) ECM array analysis of basement membrane constituents. A panel of basement membrane proteins was analyzed for expression changes with KLK5 stable transfection compared to mock. There was a general down-regulation of basement membrane constituents mRNA expression with KLK5 including the Laminin protein family (p < 0.001). (C) ECM array analysis of collagens. There was down-regulation of a large set of collagens including Col 4A2, Col 6A1, Col 5A2, and Col 5A1 with KLK5 expression. Col 8A1, Col 12A1, and Coll 11A1 had increased mRNA expression (p < 0.001). (D) ECM array analysis of extracellular matrix proteases. A set of extracellular matrix proteases was analyzed for expression changes with KLK5 stable transfection as compared to mock. There was a generalized down-regulation of the ECM Proteases including MMP15, MMP9, and MMP2 with KLK5 expression (p < 0.001).

Extracellular matrix (ECM) gene expression changes were among the most significantly affected in our prediction analysis. We identified a miRNA network with convergent (where multiple miRNAs can target the same gene) and divergent (where the same miRNA can target multiple genes along the same pathway) properties (Figure 2). To validate the proposed miRNA-mediated effect of KLK5 on ECM, an ECM profiling array was conducted which indicated significant expression alterations in ECM pathway genes in KLK5-transfectants (Figure 3). These changes included downregulation of cell to matrix adhesion molecules such as reduced integrin (ITG) expression including integrin beta-1 (ITGB1) (Figure 3A); decreased expression of basement membrane constituents (Figure 3B); reduced collagen and structural constituents expression (Figure 3C); and lower ECM proteases expression (Figure 3D).

### miRNA-ECM interactions in MDA-MB-231 cell lines

We next investigated the effect of KLK5-induced miRNAs on ECM. We were able to identify several statistically significant conserved miRNA binding sites in the 3′ UTR of the ITGB1 gene as well as other ECM genes, as shown in Supplementary Table 3.

**Figure 4:**
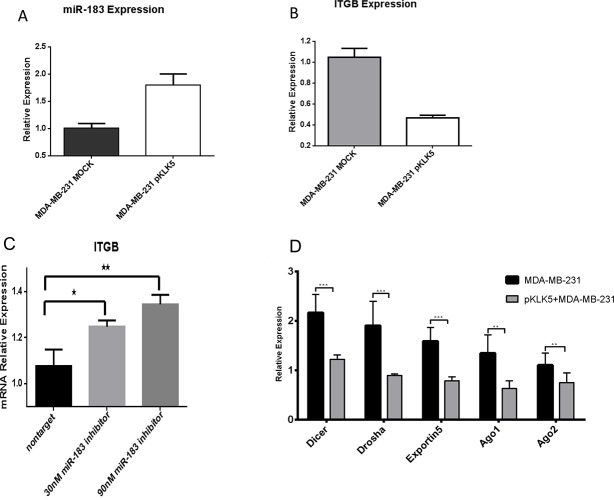
(A) KLK5 overexpression resulted in miR-183 upregulation KLK5 stably transfected clones showed significant up-regulation of miR-183 (p=0.0033). (B) Quantitative PCR analysis showed significant ITGB down-regulation in the MDA-MB-231 breast cancer cell lines transfected with KLK5 compared to controlled cells (p = 0.0004). (C) Transfection with an inhibitor of miR-183-5p resulted in reversal of the inhibition of ITGB1 confirming the effect is through inhibition by a miR-183-5p miRNA pathway to the 3′UTR miRNA 183-5p binding sites of ITGB1. Expressions are performed in triplicate and an average was calculated. p=0.0174 (nontarget and 30nM) p=0.0047 (nontarget and 90 nM). (D) The effect of KLK5 transfection on the expression of key enzymes involved in miRNA biogenesis. Clones that were stably transfected with KLK5 showed a significant down-regulation of miRNA-biogenesis enzymes (Drosha, Dicer, Exportin5, Ago 1 and Ago2) compared to the paternal control cells p= 0.0005 (Dicer), p=0.0010 (Drosha), p=0.0003 (Exportin 5), p=0.0016 (Ago1) & p=0.0088 (AGO2). All expression levels were normalized to a housekeeping gene and they are shown as relative expression values to the MDA-MB-231 mock (vector-transfected)cells (with expression value equal to 1).

In order to experimentally validate the KLK5/miR-183-5p/ITGB1 interaction, we compared miR-183-5p and ITGB1 mRNA expression in KLK5 stably transfected MDA-MB-231 cells compared to controls. Upon KLK5 transfection, there was significant elevation of miR-183-5p levels (Figure 4A). In the same time, KLK5 also resulted in significant (3-fold) reduction of ITGB1expression compared to mock cells (Figure 4B).

We hypothesized that the effect of KLK5 on ITGB1 expression is mediated through the up-regulation of miR-183-5p. In order to test this hypothesis, a miR-183-5p inhibitor was transfected into stable KLK5 expressing cells. Transfection with the inhibitor of miR-183-5p resulted in reversal of the inhibition of miR-183-5p on ITGB1 gene expression in a dose-dependent manner (Figure 4C).

### Mechanisms underlying the KLK5 effect on miRNAs

To further explore the putative mechanisms by which KLK5 can affect miRNA expression and function, we examined the expression of a number of molecules involved in miRNA biogenesis (Figure 4D). We demonstrate that KLK5 re-expression down-regulates multiple key members of the miRNA biogenesis machinery including the RNAse III-type protein and Drosha, which is involved in the initial steps of the miRNA biogenesis pathway. Furthermore, KLK5 was demonstrated to reduce Exportin5 expression, which will lead to inhibited export of miRNAs from the nucleus to the cytoplasm. Finally, KLK5 was also found to reduce the mRNA levels of RNAse III enzyme Dicer and the Argonaute (Ago) proteins (1 and 2). Dicer and Argonaute are vital for miRNA maturation for the binding of the miRNA to the mRNA complex of the target, and silencing of the mRNA through RISC.

These results indicate that the effect of KLK5 on miRNAs is mediated in part by reducing the expression of the genes involved in miRNA biogenesis. This matches the trend towards global down-regulation of miRNAs. Interestingly, using miRWALK analysis, we were able to identify a number of statistically significant miRNA response elements in the 3′ UTR of the genes coding for the enzymes involved in miRNA biogenesis, as shown in Supplementary Table 3. Some of these miRNA sites have been validated previously including miR-183: DICER1, AGO1 (EIF2C1) [20], AGO2 (EIF2C2) [21,22,23]; and miR-206: Exportin5 [24,25]. Taken together, out data points out that KLK5 re-expression in non-expressing breast cancer cells results in up-regulation of specific miRNAs which can target critical molecules involved in miRNA biogenesis with subsequent global down-regulation of miRNA expression.

### Association of KLK5 and miR-183 with biological characteristics of breast cancer

**Figure 5:**
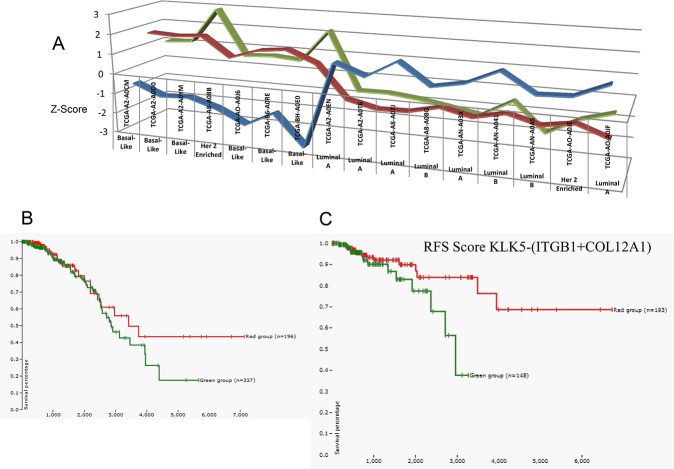
(A) Subset of patients from the TCGA database demonstrating a significant correlation between KLK5 expression and miR-183-5p expression levels, and their effects on ITGB1 expression We plotted the expression of these genes and also the breast cancer subtype. The data demonstrated a trend in which high expression of KLK5 (red) is associated with high miR-183-5p (green), low ITGB1 expression (blue) and a basal-like breast cancer subtype.Z-score from 746 breast cancer patients from TCGA [68]. (B) Overall survival (OS) with KLK5 expression in breast cancer patients TCGA and IlluminaHiSeq data visualized using the Cancer Genomics Browser (n=1106 patients). Higher KLK5 expression (−1.44472 to 0.3953, shown in red) is associated with significantly higher survival compared to lower expression levels (−5.64972 to −1.52672, shown in green). (C) Recurrence Free Survival (RFS) with KLK5-(ITGB1+COL12A1) scoring expression in breast cancer patients TCGA and IlluminaHiSeq data visualized using the Cancer Genomics Browser (n=1106 patients). Green indicates score −4.87938 to −0.81838; Red indicates score of 0.87962 to 7.43662[68,67].A higher score is associated with significantly longer survival.

In order to assess potential clinical implications of the KLK5-miR-183-5p axis in breast cancer behavior, we examined the expression of these molecules in breast cancer tissues from patients through the TCGA databases. A sub-set of patients were recovered with changes in KLK5, miR-183-5p and ITGB1 expression of at least 1 z-score. Higher expression of KLK5 correlated with higher levels of miR-183-5p, lower ITGB1 expression, and a basal-like breast cancer subtype (Figure 5A). These findings further confirm our proposed KLK5-miR-183-5p-ITGB1 axis of interaction. Lower expression of KLK5 and miR-183-5p was correlated with higher ITGB1 expression and a luminal A or B breast cancer subtype in these patients. Interestingly, demonstrated metastasis was only found in the two patients with the lowest expression of KLK5 and miR-183-5p, and the highest ITGB1 expression (Supplementary Figure 1).

### Potential prognostic significance of KLK5

Examination of survival data in our dataset (IlluminaHiSeq, n=1106) demonstrated higher long-term overall survival in patients with basal-like breast cancer, compared with luminal B and Her-2 enriched, as expected. Overall survival was significantly shorter in association with lower KLK5 expression levels (Figure 5B). Moreover, we developed a predictive score KLK5-(ITGB1+COL12A1) which demonstrated patients with the highest scores had improved long-term recurrence free survival (RFS) compared with patients with the lowest scores (IlluminaHiSeq, n=1106) (Figure 5C).

### Clinical Validation of KLK5-miRNA axis in breast cancer subtypes

**Figure 6:**
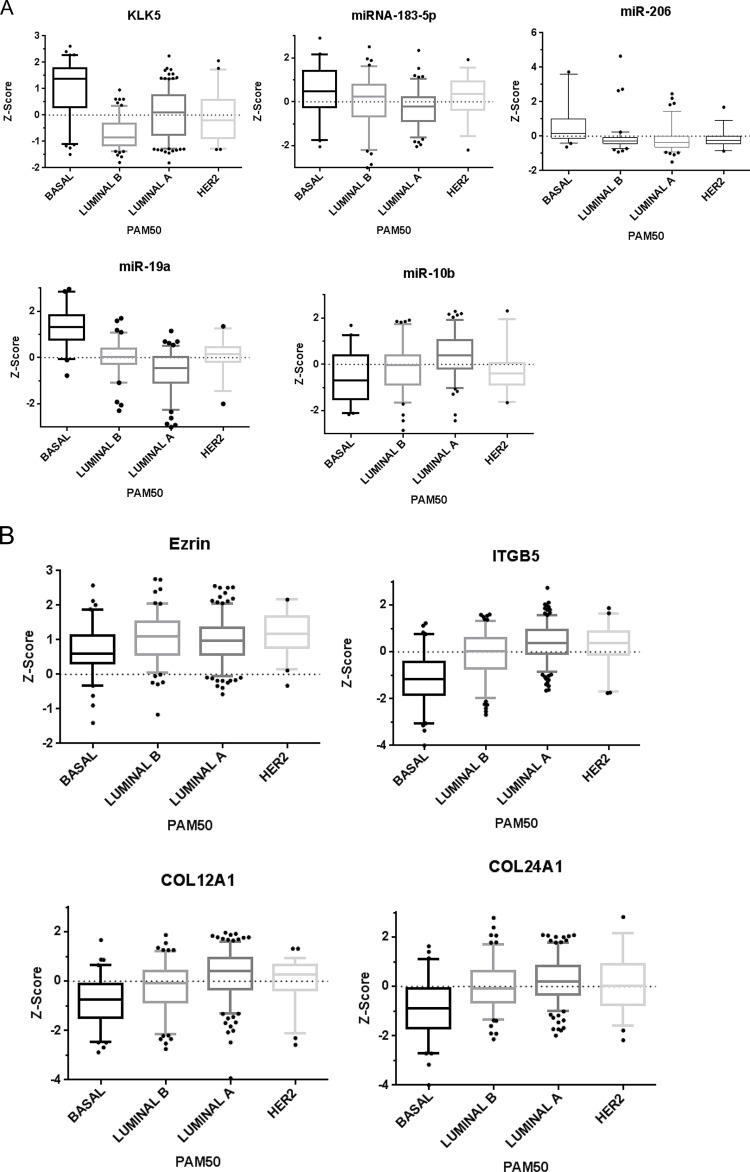

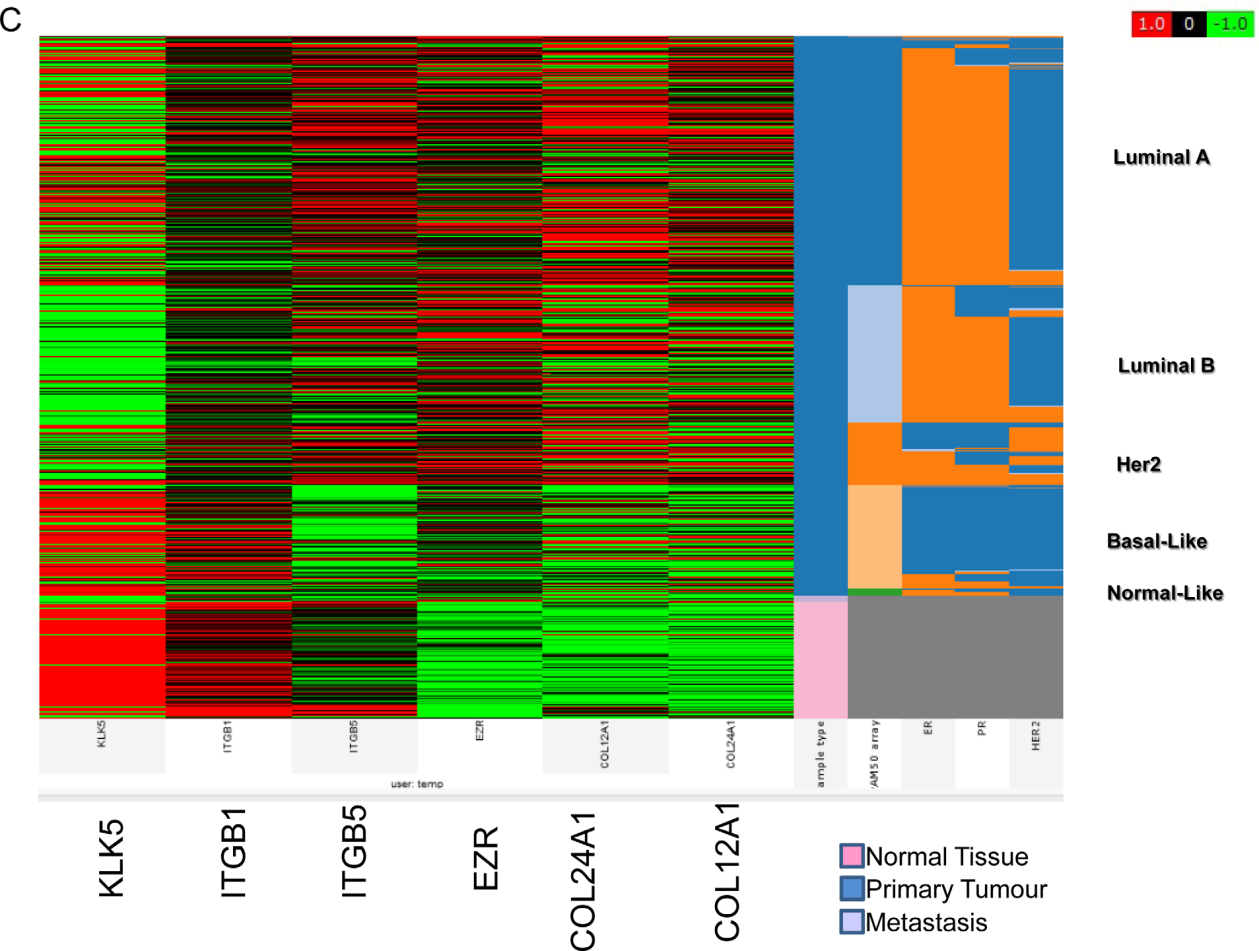
(A) KLK5 and KLK-regulated miRNA expression in invasive breast cancer subtypes Expression of KLK5 is significantly elevated in basal-like human breast cancers as compared to luminal B breast cancer subtypes. miR-183-5p, miR-206 and miR-19a expressions are significantly elevated in basal-like human breast cancers as compared to luminal B breast cancer subtypes, whereas miR-10b expression is significantly reduced in basal-like as compared to luminal B human breast cancer subtypes (p<0.0001). Data analyzed from TCGA [68]. 81 basal-like 129 luminal B, 232 luminal A, and 58 Her's-2 breast cancers. Data plotted as mean box & whisker plots, 5-95% CI with outliers shown. (B). KLK5 and KLK-regulated mRNA expression in invasive breast cancer molecular subtypes. A number of miRNA-mediated KLK5-regulated genes were found to have an inverse correlation with KLK5 expression in breast cancer subtypes. Expression of Ezrin is significantly reduced in basal-like human breast cancers as compared to luminal B breast cancer subtypes (A). ITGB1 expression is elevated in basal-like human breast cancers as compared to luminal B breast cancer subtypes (B). ITGB5 expression is significantly reduced in basal-like human breast cancers (Mean −0.8075) as compared to luminal B breast cancer subtypes (Mean −0.2960) (C). COL12A1 expression is significantly reduced in basal-like human breast cancers as compared to luminal B breast cancer subtypes (D). Similarly, COL24A1 expression is significantly reduced in Basal-like human breast cancers as compared to luminal B breast cancer subtypes (E). Data analyzed from TCGA [68]. 81 basal-like, 129 luminal B, 232 luminal A, and 58 Her-2 breast cancers. (p<0.0001). Data plotted as mean box & whisker plots, 5-95% CI with outliers shown. (C). Microarray visualization of KLK5 and KLK-regulated mRNA expression in invasive breast cancer molecular subtypes. IlluminaHiSeq data visualized using the Cancer Genomics Browser, n=1106 patients. Red indicates gene expression of +1 Z-score, green indicates −1 Z-score gene expression, and black indicates 0 Z-score. [68], [67]. Data plotted as Box and Whiskers Plot (5-95% with outliers plotted), data analyzed from TCGA. 81 basal-like, 129 Luminal B, 232 luminal A, and 58 Her-2 breast cancers.

Analysis of TCGA data validated the link between the identified KLK5-miRNA axis and breast cancer subtypes. Using over 500-patient samples comprised of 80 basal-like, 128 luminal B, 231 luminal A, and 57 Her2 overexpressing breast cancers, we found that KLK5 expression was significantly reduced in the luminal B subtype as compared with basal-like breast cancers (Figure 6A). Luminal B is known to have aggressive behavior including invasion and metastasis. Expression of miRNA-183 positively correlated to KLK5 expression, with the highest miR-183 levels in basal-like breast cancers (Figure 6A). This further supports that KLK5 expression is correlated to miRNA network regulation. Also, miR-206 and miR-19a were also demonstrated to have a positive correlation with KLK5 levels with elevated expression in basal-like breast cancers, whereas miR-10b expression was inversely correlated to KLK5 expression with the lowest levels in patients with basal-like breast cancers (Figure 6A). To further confirm our data, we visualized a second clinical dataset (IlluminaHiSeq, n=1106), which demonstrated low miR-10b in basal-subtypes (Supplementary Figure 2). Interestingly, miR-19a expression positively correlated with KLK5 expression with higher expression in basal-like subtypes. Other highly up-regulated or down-regulated miRNA data was unfortunately not available to assess visually with this microarray data set. Taken together, KLK5 expression and its effects on miRNA networks and the ECM molecule expression may provide a contributing mechanism to luminal B subtypes' aggressive behavior.

### Clinical Validation of miRNA-ECM Targets in breast cancer subtypes

In order to validate miRNA network regulation of ECM genes, we assayed the TCGA database. Ezrin, a validated target of miR-183 was found to be negatively correlated to KLK5 expression with lower expression in basal-like and higher expression in luminal B subtypes (Figure 6B). Also, a number of other targets of the up-regulated miRNAs, including ITGB5, COL12A1, and COL24A1 expression were found to be lower in basal-like compared to the other subtypes (Figure 6B). The correlation between ITGB1 and KLK5 expression was also noted in a subset of patients (Figure 5A), but was not significant in the larger cohort of patients, indicating the presence of other mechanisms that control ITGB1 expression.

To further confirm our data, we visualized a second clinical dataset (IlluminaHiSeq, n=1106), which demonstrated higher expression of KLK5 in normal tissue, with a reduction of expression in metastatic tissues (Figure 6C). Interestingly, expression of KLK5 was high in basal-like and normal-like breast cancer subtypes with a reduction in levels in luminal B. There was reduced expression in normal tissue and basal subtypes with higher expression in luminal (ER+) and Her-2 subtypes for ITGB5, EZR, COL12A1, and COL24A1. Changes in ITGB1 expression were not significant.

We further validated the expression of other ECM genes in the different subtypes through the TCGA database. MMP 2, 11, 13negatively correlated to KLK5 expression with reduced expression in basal-like and elevated levels in luminal B subtypes (Supplementary Figure 3). MMP3 expression did not show significant changes. We also visualized a second clinical dataset (IlluminaHiSeq by RNASeq, n=1106), which demonstrated lower expression of all MMPs examined with the exception of MMP2 in normal tissue. Interestingly, expression of MMPs 3, 11, and 13 were negatively correlated to KLK5 expression with the highest expression levels in the Her-2 and luminal groups (Supplementary Figure 4).

### Proposed model for a KLK5-miRNA-ECM network in breast cancer

**Figure 7:**
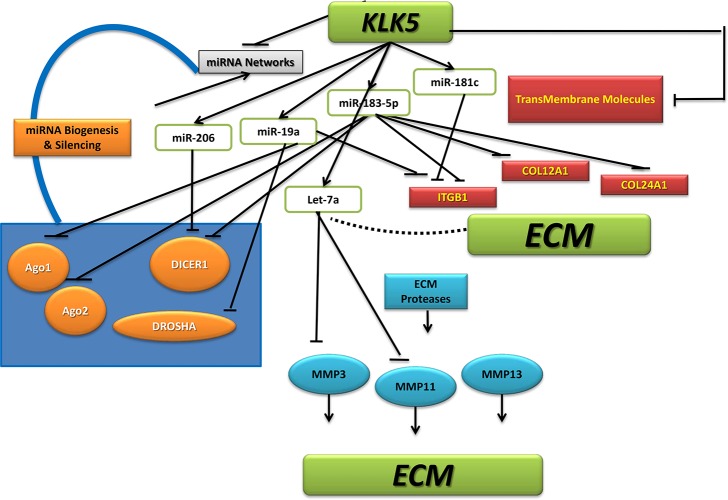
Proposed model for a KLK5-miRNA-ECM network in breast cancer KLK5 regulates ECM and cell adhesion both directly through its proteolyitc activity, and through miRNA-mediated pathways. KLK5 overexpression results in up-regulation of a subset of miRNAs and down-regulation of another miRNA subset. miRNA under-expression can be mediated through affecting critical enzymes involved in miRNA biogenesis which in turn can be mediated through the up-regulation of a number of miRNAs that are known to target these enzymes. These pathways merge to have an amplified downstream impact on ECM regulation, breast cancer phenotype and metastatic potential.

Our data demonstrates that KLK5 expression affects the expression of a network of interacting miRNAs. These miRNA networks were found to be enriched in ECM molecule targets. We propose a model in which KLK5 not only regulates ECM and cell adhesion directly post-translationally through its proteolyitc activity, as previously reported [26], but also affects the level of these proteins through miRNA-mediated pathway(s). KLK5 over-expression results in up-regulation of a subset of miRNAs and also to down-regulation of another miRNA group. miRNA under-expression can be mediated through affecting critical enzymes involved in miRNA biogenesis which in turn can be mediated through the up-regulation of a number of miRNAs that are known to target these enzymes. These pathways merge to have an amplified downstream impact on ECM regulation, breast cancer phenotype and metastatic potential (Figure 7).

## DISCUSSION

In line with recent literature documenting the critical involvement of miRNAs in oncogenesis and their role in breast cancer progression [27], our results add a new dimension by demonstrating a crosstalk between proteolysis and miRNAs, and a potential regulatory role of KLK5 in controlling the expression of miRNA networks which affect target ECM molecule interactions.

The potential utility of KLK5 as a cancer biomarker has been reported. Reduced expression of KLK5 has been observed in breast cancers[9,28], and in malignant compared to benign breast tissue [29]. KLK5 is also found to be down-regulated in pancreatic [30], prostate [31], lung [32], testicular [33], and renal cancers [34], pointing to a tumor suppressor function. We demonstrated that KLK5, and a combined KLK5 scoring that includes ECM genes can predict breast cancer patient survival as well the differentiating between breast cancer subtypes.

Luminal breast cancers are highly correlated to estrogen receptor (ER) positive expression (82% of luminal B and 87% luminal A *versus* 10% ER positivity in basal-like and 20% positivity in Her-2 type [35]. Reduced KLK5 expression was previously found to correlate with ER-negative breast cancers which are mainly composed of the subtypes luminal A and B. Of 102 samples, 77% patients which were ER-positive were also KLK5-negative [29]. This is in keeping with our findings where the lowest KLK5 expression was in the ER+ luminal B subtype compared to other subtypes.

Reduced KLK5 expression as found in luminal B and Her-2 subtypes corresponded to reduced overall survival. Similarly, the lowest composite scores of KLK5-ITGB1-COL12A1 had the poorest RFS as compared to the highest scores. Our hypothesis and data is, therefore, in line with the U133A tumor compendium dataset of 1,340 breast cancer patients which demonstrated luminal B has the worst prognosis in terms of distant metastasis-free survival over 25 years, followed by luminal A, Her-2 and basal-like [36].

We also provide evidence that the KLK5-miRNA-ECM axis could be related to the biological differences between the breast cancer subtypes. KLK5 affected the expression of a network of miRNAs, which are known to inhibit invasion and metastasis in breast cancer. We confirmed these results both experimentally by reconstituting KLK5 expression in cell lines [37], and clinically by showing a correlation between KLK5 expression and a number of these miRNAs in high metastatic potential cancers such as luminal B and Her-2 enriched subtypes. Up-regulated miRNAs included miR-19a, which is known to inhibit tissue factor. Tissue factor regulates tumor angiogenesis, metastasis, and is selectively expressed in highly invasive cancer cells [38]. In the study by Tavazoie et al., miR-335 was demonstrated to inhibit metastasis and regulate genes associated with risk of distal metastasis. Furthermore, expression of miR-335 is lost in the majority of primary breast tumors from patients who relapse [39].

We also show that miR-10b, a pro-metastatic miRNA, was reduced upon KLK5 overexpression, and was also lower in the basal-like subtype that has higher KLK5 expression. miR-10b was demonstrated to be highly expressed in about 50% of metastatic tumors [40]. In addition, miR-10b is over-expressed by 50-fold in metastatic MDA-MB-231 cell lines, in comparison with non-metastatic MCF7 cells [41]. Our data suggest that miR-10b can be a downstream effector target of KLK5.

KLK5 demonstrated higher expression in the basal-like subtype, with positive correlation with miR-206 and miR-183-5p. miR-206 is known to be down-regulated in ER-positive breast cancers, and it inhibits cell growth in a dose-dependent manner[42]. Literature also describes miR-206 as a tumor suppressor [40]. In an analysis comparing invasive to less invasive cell lines, miR-183 was demonstrated to be down-regulated in invasive cell lines including MDA-MB-231 [43]. Furthermore, miR-183-5p was significantly down-regulated in 8 of 11 human breast cancer stem cells [44]. It has also been demonstrated that over-expression of miR-183 inhibits migration of cancer cells through its action on VIL2 / Ezrin [45]. In other cancers, miR-183 has also been demonstrated to reduce metastasis through its action on Ezrin [46].

In order for cancer cells to metastasize, they must undergo invasion, migration and attachment to a new tissue. Integrins play an important role in these processes [47]. Antagonists of several integrins (α5β1, αvβ3 and αvβ5) are currently under evaluation in clinical trials to determine their potential as therapeutics for cancer and other diseases [48]. MDA-MB-231 breast cancer cell lines express high levels of ITGA2 / ITGB1, ITGA3 / ITGB1, ITGA5 / ITGB1, ITGAV / ITGB3 integrins, compared to MCF-7, T47D, and ZR75-1 breast cancer cells [49]. ITGB1 was previously shown to be targeted by miR-183-5p. miR-183 significantly decreased the expression of ITGB1 as measured by Western blot and immunocytochemistry [50]. Furthermore Li et al. demonstrated that miR-183 transfection led to a significant decrease in the cellular invasion and migration capacities of HeLa cells [50]. Blocking ITGB1 binding activity has been shown to revert the transformed phenotype of human breast cancer cells [51,52]. Furthermore, targeted disruption of ITGB1 in a transgenic mouse model of human breast cancer inhibited both initiation and maintenance of mammary tumor growth [53]. COL12A1 has been shown to be a statistically significant representation of myoepithelial types [54], and loss of myoepithelial function is a key step in breast cancer progression[55].

Proteases and their inhibitors play important roles in tissue invasion and cell migration. KLK5 seems to play important roles in cell-to-matrix adhesions. Previous reports identified several ECM molecules including collagens type I, II, III, and IV, fibronectin, and laminins as potential KLK5 targets [26]. KLK5 has also been demonstrated to interact with α2-integrins, resulting in suppression of invasion of cancer through ectodomain regulation [30]. We reported that re-expression of KLK5 results in suppressed motility of MDA-MB-231 cells consistent with the observed down-regulation of the embryonic transcription factor SNAIL1 and reduced expression and proteolytic activity of MMP9[11]. Furthermore, KLK5 inhibits RhoA signaling by suppressing cholesterol/isoprenoid biosynthesis [11], which could account for the inhibition of MMP-9[56]. Here, EMC array analysis confirmed the anti-correlation of KLK5 and MMP9, which was found to be the most highly suppressed protease upon KLK5 re-expression. An intriguing novel finding of our present analysis is the inverse correlation of KLK5 and ezrin, a membrane cytoskeleton linker protein, which has been shown to increase tumor motility and invasion, and is considered key to the metastatic process [57].

KLKs and MMPs have been demonstrated to regulate each other post-translationally by limited proteolysis [58,59,60]. We show here that KLKs and MMPs may regulate each other also post-transcriptionally through regulatory miRNA networks. MMPs have been demonstrated to have a significant role in breast cancer invasion and metastasis due to their ability to degrade extracellular matrix proteins [61]. Here, we provide evidence that KLK5 may regulate MMPs through miRNA networks, and MMP-2, -11, -13 and their expression is lower in basal-like subtypes. It has been well-documented that cellular and ECM matrix interactions regulate downstream cellular pathways including miRNA networks, cell survival and apoptosis through pathways such as p53 [62,63]. We postulate that the protease activity of KLK5 may affect a miRNA network indirectly through its effect on the ECM and subsequent down-stream signaling pathways, which can affect transcription and miRNA processing.

In conclusion, we provide evidence for KLK5-mediated regulation of a network of miRNAs in breast cancer, with consequences on downstream ECM regulatory target gene expression linked to metastatic potential, patient survival and the molecular subtype of breast cancers with potential clinical applications.

## MATERIALS AND METHODS

### Cell culture and plasmid transfection

The MDA-MB-231 cell line was obtained from the American Type Culture Collection. Cells were cultured as described [11]. The cDNA encoding preproKLK5 was amplified by PCR from a full-length *KLK5* cDNA using gene-specific primers and cloned into the pcDNA3.1 (+) vector (Invitrogen, Carlsbad CA). Plasmids were purified (Qiagen, Valencia, CA) and confirmed by DNA sequencing (ACGT, Toronto, Canada). Stably transfected MDA-MB-231 cells were selected with G418.

### miRNA and gene expression profiling

Illumina chip assayed mRNA data was obtained as described [11]. Expression of 754 miRNAs was compared before and after KLK5 transfection using TaqMan® Low Density Arrays. RNA extraction was carried out using the miRNeasy (Qiagen). The integrity and concentration of total RNA was quantified using a bioanalyzer (Agilent). cDNA was synthesized from 200ng of total RNA using TaqMan® MicroRNA Reverse Transcription and the Megaplex™ RT Primers (Life Technologies) as per the manufacturer's instructions. 200ng total RNA was mixed with 10X Megaplex RT Primers, 100mM dNTPs, 10X RT Buffer, 25mM MgCl_2_, and 20U/μL RNase Inhibitor. Samples underwent 40 cycles of 16°C for 2 mins, 42°C for 1 min and 50°C for 1 sec. Expression was quantified using the PCR-based TaqMan® Low Density Arrays. cDNA was added to TaqMan Master mix loaded onto miRNA Arrays A and B. Arrays were run on the Applied BiosystemsViiA™ 7 real-time PCR system and data was analyzed using the Expression Suite Software. miRNA expressions on Array A were normalized with hsa-let-7d and those on Array B were normalized using hsa-miR-151-3p. Mock-transfected cells were used as a calibrator.

### miRNA target prediction

Target prediction analysis was performed using different programs including TargetScanHuman 6.2 (http://www.targetscan.org/), DIANA-mirPath (http://diana.cslab.ece.ntua.gr/pathways/), the PITA database (http://genie.weizmann.ac.il/pubs/mir07/mir07_prediction.html), MiRanda (http://www.microrna.org/microrna/home.do), DIANA-microT (http://www.diana.pcbi.upenn.edu/cgi-bin/micro_t.cgi), miRWalk [64],RNA22 (http://cbcsrv.watson.ibm.com/rna22.html), and miRDB (http://mirdb.org/miRDB/). Only predictions of at least three independent programs were included for analysis.

### Experimental target validation

Predicted gene targets were confirmed to have altered expression in *KLK5*-expressing stably transfected clones compared to controls by mRNA microarray expression analysis. PCR analysis using gene-specific probes were used for independent validation. miRNA expressions were assessed by qRT-PCR using TaqMan® probes, as in our recent studies [65].

### Pathway enrichment analysis

Pathway enrichment analysis was performed using DIANA miRPath v2.1 [66]. Enrichment analysis software (http://diana.cslab.ece.ntua.gr/) was employed to gain insight into global molecular networks and canonical pathways related to differentially expressed miRNAs between *KLK5*-expressing and non-expressing MDA-MB-231 cell lines. miRPath identified multiple miRNA target genes using enrichment analysis by comparing each set of miRNA gene targets to all known KEGG pathways. Pathways showing p-value <0.05 were considered significantly enriched pathways after analysis.

### Analysis of ECM gene expression by quantitative RT-PCR

Cells were homogenized, and total RNA was extracted according to RNAEasy standard protocol (RNAEasy, Qiagen). RNA purity and quality were assessed using a Bioanalyzer (Agilent Technologies). A260/A230 ratio was over 1.7, and A260/A280 ratio was greater than 2.0. Gene expression was assessed by qRT-PCR using TaqMan® probes and the Human Extracellular Matrix and Adhesion Molecules array (RT2 Profiler PCR Array PAMM-013; SuperArray Biosciences, Frederick, MD). Gene expression was compared between MDA-MB-231transfectants and mock controls. Genes with 2-fold change and p< 0.05 were identified.

### miRNA loss of function experiments

Anti-miR-183-5p were used to knockdown miR-183 (Life Technologies). Cells were transfected with either 30nM or 90nM of specific or non-targeting anti-miR using siPORT NeoFX transfection agent (AM4510) for 24h before used for subsequent experiments.

### Breast cancer clinical data validation

We validated our results on an independent set from the Cancer Genome Atlas (TCGA) databases [2]. The breast cancer (BRCA) data set was downloaded from TCGA public data portal, and from the cBio Cancer Genomics Portal at the Memorial Sloan-Kettering Cancer Center. Data was analyzed from over 825 breast cancer cases, which comprised tumor mRNA array expression data (n=526) and tumor miRNA array expression data (n=302). For expression data analysis, z-scores or “level 3” normalized data from mRNA and miRNA microarrays were used. Molecular subtype data included luminal B (n=131), luminal A (n=235), basal-like (n=80), and Her-2 enriched (n=58). For microarray analysis, we employed the IlluminaHiSeq expression data set (n=1106) using the Genomics Browser to visualize the data[67].

## SUPPLEMENTARY MATERIAL TABLES AND FIGURES



## Abbreviations

KLK: Kallikrein-related peptidase
miRNA: microRNA
TCGA: the cancer genome atlas
ECM: Extracellular matrix
ITGA: Integrin alpha
ITGB: Integrin beta
COL12A1: Collagen alpha-1
PCR: Polymerase chain reaction
qRT-PCR: quantitative Reverse Transcription Polymerase Chain Reaction
KEGG: Kyoto Encyclopedia of Genes and Genomes
3′UTR: three prime untranslated region
AGO: Argonaute
MMP: Matrix metalloproteinase
ER: Estrogen receptor
