# Supplementary material for: Kallikrein-related peptidase 5 induces miRNA-mediated anti-oncogenic pathways in breast cancer
Source: Oncoscience. 2014 Oct 24;1(11):709–24. doi: 10.18632/oncoscience.91 (PMC4278268; doi:10.18632/oncoscience.91)
Supplement: Supplementary file 1 [file oncoscience-01-0709-s001.pdf]

Kallikrein-related peptidase 5 induces miRNA-mediated anti-oncogenic pathways in breast cancer

Supplementary Material

Supplementary Table 1: Enriched miRNA target pathways in KLK5 reconstituted expressing breast cancer cells.

**Down-regulated miRNAs**				**Up-regulated miRNAs**			
**KEGG Pathway**	**p-value**	**# genes**	**# miRNAs**	**KEGG Pathway**	**p-value**	**# genes**	**# miRNAs**
MAPK signaling	0.004723	132	44	Glycosaminoglycan biosynthesis - heparan sulfate	1.15E-13	10	13
Apoptosis	0.040558	43	37	TGF-beta signaling pathway	4.83E-06	45	18
p53 signalling	0.045807	37	31	Arrhythmogenic right ventricular cardiomyopathy (ARVC)	1.24E-05	36	18
Oocyte meiosis	0.002479	65	42	Endocytosis	1.52E-05	90	20
Osteoclast differentiation	0.045807	63	42	ECM-receptor interaction	1.52E-05	35	15
Vascular smooth muscle contraction	0.039203	58	39	Glioma	8.67E-05	32	17
T cell receptor signaling	0.049869	55	39	Axon guidance	0.00018	57	20
Amoebiasis	0.045807	50	39	Regulation of actin cytoskeleton	0.00107	93	19
mRNA surveillance	0.008913	48	38	Adherens junction	0.0039	38	14
Gastric acid secretion	0.008913	43	39	Pathways in cancer	0.0054	135	21
Calcium signalling	0.007937	89	46	Focal adhesion	0.0054	85	19
Renal cell carcinoma	0.007038	38	38	Wnt signaling pathway	0.0057	66	18
Protein processing in endoplasmic reticulum	0.000495	89	43	Neurotrophin signaling pathway	0.0068	57	19
Hedgehog signaling	0.022192	32	37	ErbB signaling pathway	0.0068	43	18
Basal cell carcinoma	0.039203	31	30	Salivary secretion	0.0068	42	17
Vasopressin-regulated water reabsorption	0.034011	29	25	Long-term potentiation	0.0068	35	17
Prion diseases	1.48E-09	19	24	GnRH signaling pathway	0.0384	43	19
D-Glutamine and D-	0.02659	4	5	Ubiquitin mediated	0.0092	59	19

glutamate metabolism	7			proteolysis			
Fatty acid biosynthesis	0.003685	1	1	Melanogenesis	0.0190	44	17

Supplementary Table 2: Combined (up and downregulated) miRNA target pathways in KLK5 reconstituted expressing breast cancer cell lines.

KEGG Pathway	p-value	# genes
Pathways in cancer	<0.0001	70
Endocytosis	<0.0001	65
Ubiquitin mediated proteolysis	<0.0001	62
Focal adhesion	<0.0001	63
Wnt signaling pathway	<0.0001	63
Regulation of actin cytoskeleton	<0.0001	64
Neurotrophin signaling pathway	<0.0001	60
Insulin signaling pathway	<0.0001	60
Salivary secretion	<0.001	56
ErbB signaling pathway	<0.001	61
Pancreatic cancer	<0.001	59
Prostate cancer	<0.001	53
Melanogenesis	<0.001	53
Adherens junction	<0.001	56
Long-term potentiation	<0.001	52
Glioma	<0.001	59
TGF-beta signaling pathway	<0.001	57
Gap junction	<0.001	49
mTOR signaling pathway	<0.001	48

Supplementary Table 3: miRNA binding sites of selected miRNA target genes.

miRNA	Gene	RefSeqID	Seed Length	Start	Sequence	End	p-value	SPMS*
miR-1301	COL12A1	NM_080645	8	7468	UUGCAGCU	7461	0.0333	1
miR-1301	COL12A1	NM_004370	8	10960	UUGCAGCU	10953	0.0333	1
miR-183	COL12A1	NM_080645	8	6961	AUGGCACU	6954	0.0333	2
miR-183	COL12A1	NM_080645	8	6869	AUGGCACU	6862	0.0333	2
miR-183	COL12A1	NM_004370	8	10453	AUGGCACU	10446	0.0333	2
miR-183	COL12A1	NM_004370	8	10361	AUGGCACU	10354	0.0333	2
miR-25	COL12A1	NM_080645	8	6817	CAUUGCAC	6810	0.0333	1
miR-25	COL12A1	NM_004370	8	10309	CAUUGCAC	10302	0.0333	1
let-7 a*	COL24A1	NM_152890	8	5678	GAGGUAGU	5671	0.0198	2
miR-183	COL24A1	NM_152890	8	6065	UAUGGCAC	6058	0.0198	2
miR-206	DICER1	NM_030621	9	6681	GGAAUGUAA	6673	0.0163	1
miR-206	DICER1	NM_177438	9	6737	GGAAUGUAA	6729	0.0163	2
miR-19a	DROSHA	NM_001100412	8	5372	AGUUUUGC	5365	0.0142	2
miR-19a	DROSHA	NM_013235	8	5407	AGUUUUGC	5400	0.0142	2
miR-19a	EIF2C1	NM_012199	11	4837	GUUUUGCAUAG	4827	0.0011	2
miR-183	EIF2C2	NM_012154	8	3300	AUGGCACU	3293	0.0143	2
miR-335	EZR	NM_001111077	8	2726	UUUUUCAU	2719	0.0181	1
miR-335	EZR	NM_003379	8	2760	UUUUUCAU	2753	0.0181	1
miR-183	EZR	NM_001111077	8	2317	UAUGGCAC	2310	0.0181	1
miR-183	EZR	NM_003379	8	2351	UAUGGCAC	2344	0.0181	1
miR-181c	ITGB1	NM_033666	8	2801	ACAUUCAA	2794	0.0154	2
miR-183	ITGB1	NM_002211	9	3637	UAUGGCACU	3629	0.0048	1
miR-183	ITGB1	NM_033667	9	3532	UAUGGCACU	3524	0.0049	1
miR-183	ITGB1	NM_033668	9	3497	UAUGGCACU	3489	0.0051	1
miR-183	ITGB1	NM_033669	9	3514	UAUGGCACU	3506	0.0049	1
miR-183	ITGB1	NM_133376	9	3552	UAUGGCACU	3544	0.0048	1
miR-19a	ITGB1	NM_033666	8	3024	AGUUUUGC	3017	0.0154	1
miR-19a	ITGB5	NM_002213	7	3203	UGUGCAA	3197	0.0415	1
miR-335	ITGB5	NM_002213	10	3308	UUUUCAUUAU	3299	0.0007	2
miR-335	ITGB5	NM_002213	7	2739	UUUUCAU	2733	0.0415	2
miR-449b	MMP2	NM_001127891	8	2187	AGGCAGUG	2180	0.019	1
miR-449b	MMP2	NM_004530	8	2334	AGGCAGUG	2327	0.019	1
miR-10b*	MMP3	NM_002422	8	1643	ACAGAUUC	1636	0.005	1
miR-520d-3p	MMP3	NM_002422	7	1533	AAAGUGC	1527	0.0199	1
miR-888	MMP3	NM_002422	7	1817	ACUGACA	1811	0.0199	2
miR-519a	MMP3	NM_002422	7	1533	AAAGUGC	1527	0.0199	1
let-7a*	MMP11	NM_005940	7	2111	UGAGGUA	2105	0.0469	1
miR-1301	MMP11	NM_005940	7	2102	UUGCAGC	2096	0.0469	1
let-7a*	MMP13	NM_002427	8	2372	CUAUACAA	2365	0.0193	1

* Starting Position of miRNA Seed Sequence. EIF2C1 =AGO1, EIF2C2 = AGO2.

Supplementary Figure 1

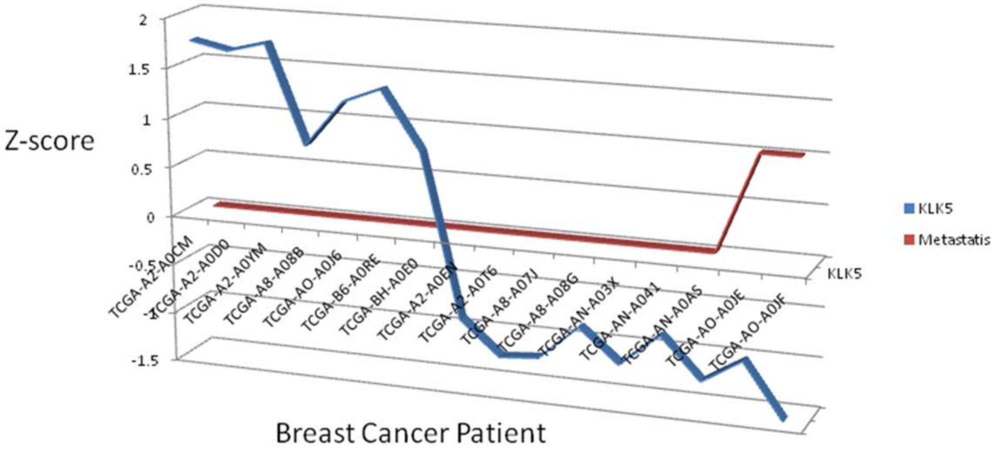

Supplementary Figure 1:KLK5 expression and metastatic potential. KLK5 expression plotted as z-score of 746 TCGA breast cancer patients – subset of 16 patients. Metastasis is shown as an absolute value (0 is no metastasis, 1 is metastasis was present as shown by sentinel node biopsy confirmed in the pathology reports). In this subset of patients, metastasis is demonstrated only in two of the lowest z-score KLK5 expressing patients.

Supplementary Figure 2

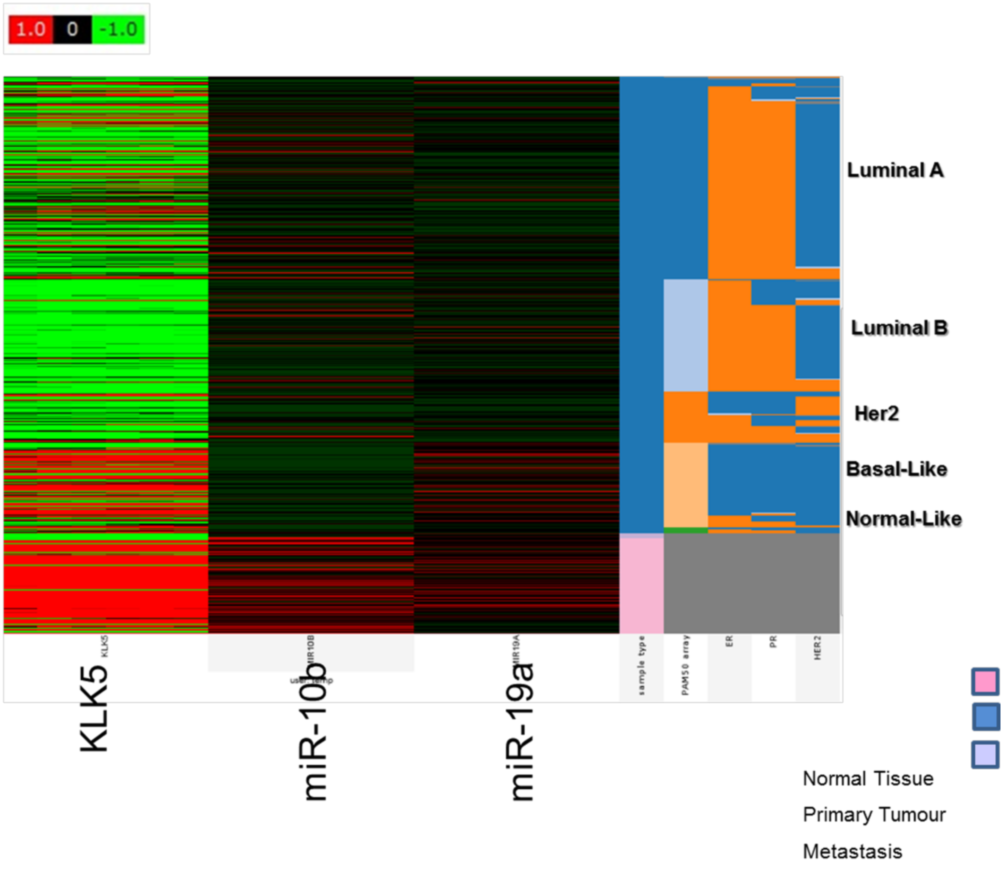

Supplementary Figure 2: IlluminaHiSeq data visualized using the Cancer Genomics Browser, n=1106 patients. Red indicates gene expression of +1 Z-score, green indicates -1 Z-score gene expression, and black indicates 0 Z-score [68],[67].

Supplementary Figure 3

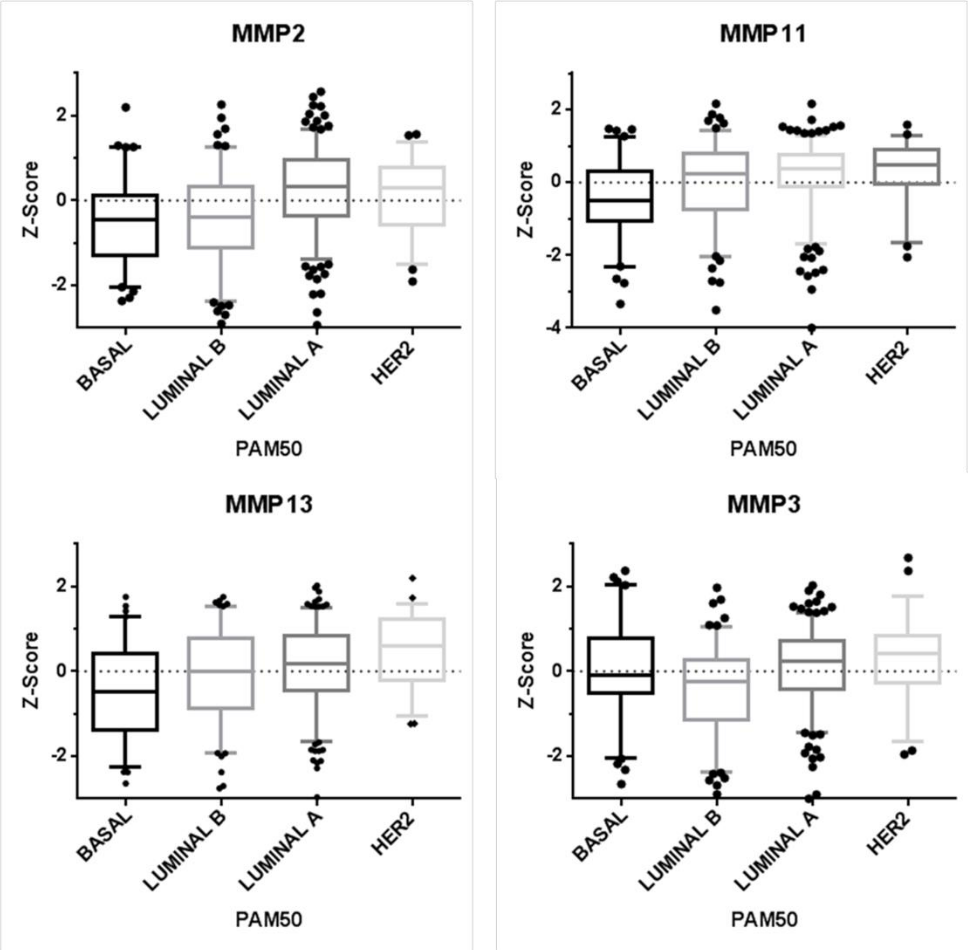

Supplementary Figure 3: KLK regulated MMP mRNA expression in invasive breast cancer subtypes. Expression of MMP13, MMP2 and MMP11 are significantly reduced in basal-like human breast cancers as compared to luminal B breast cancer subtypes. MMP3 mRNA expression was increased in basal-like as compared to luminal B human breast cancers. Data plotted as Box and Whiskers Plot (5-95% with outliers plotted), data analyzed from TCGA [68]. 81 basal-like, 129 luminal B, 232 luminal A, and 58 Her-2 Breast cancers.

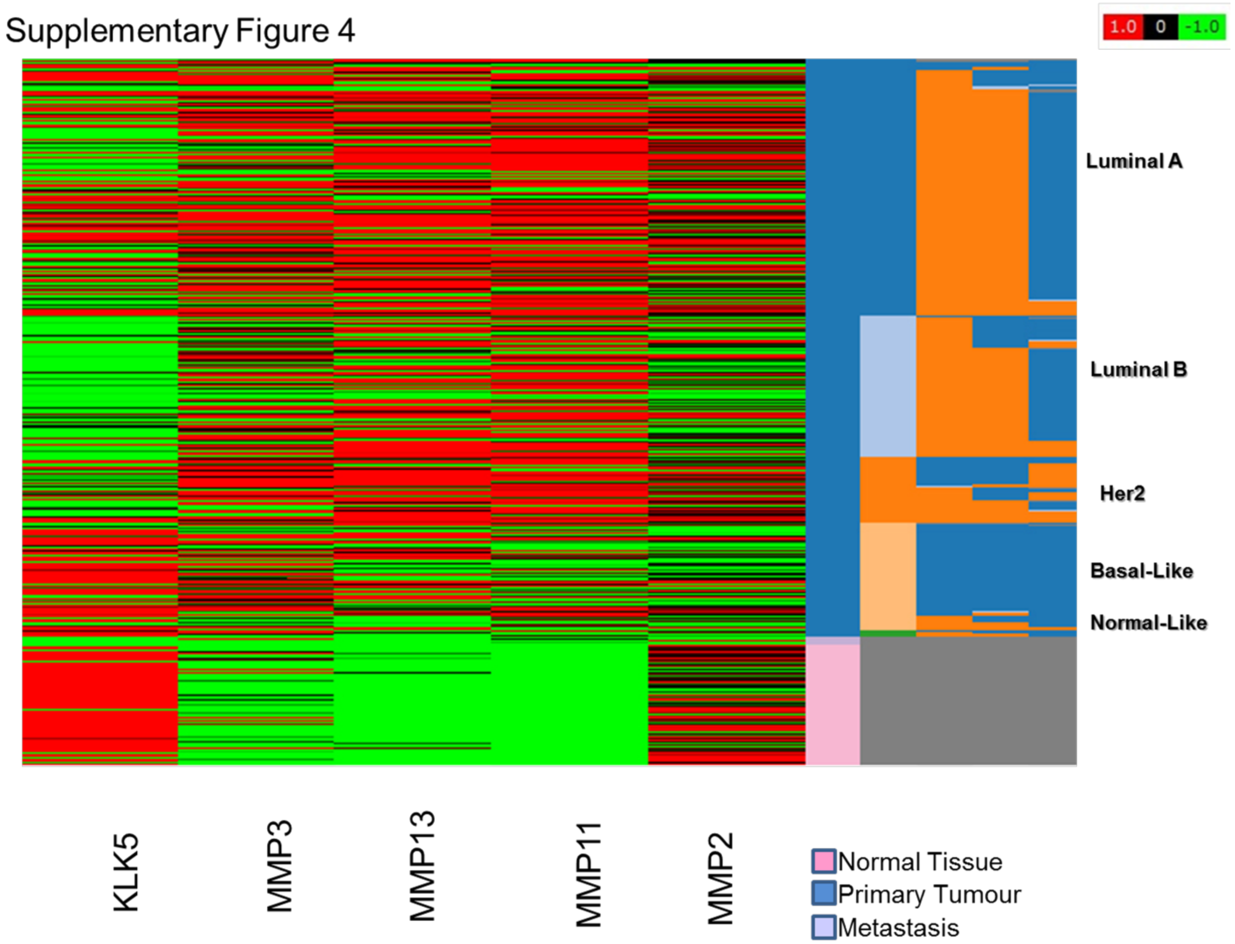

Supplementary Figure 4: Microarray visualization of MMP mRNA expression in invasive breast cancer subtypes. IlluminaHiSeq data visualized using the Cancer Genomics Browser, n=1106 patients. Red indicates gene expression of +1 Z-score, Green indicates -1 Z-score gene expression, and black indicates 0 Z-score [67].
